# Evaluation of a national cryptococcal antigen screening program for HIV-infected patients in Uganda: A cost-effectiveness modeling analysis

**DOI:** 10.1371/journal.pone.0210105

**Published:** 2019-01-10

**Authors:** Radha Rajasingham, David B. Meya, Gregory S. Greene, Alexander Jordan, Mina Nakawuka, Tom M. Chiller, David R. Boulware, Bruce A. Larson

**Affiliations:** 1 Division of Infectious Diseases & International Medicine, University of Minnesota, Minnesota, United States of America; 2 Infectious Diseases Institute, Makerere University, Kampala, Uganda; 3 Mycotic Diseases Branch, Centers for Disease Control, Atlanta, Georgia, United States of America; 4 AIDS Control Program, Ministry of Health, Kampala, Uganda; 5 Department of Global Health, Boston University School of Public Health, Boston, Massachusetts, United States of America; Gettysburg College, UNITED STATES

## Abstract

**Background:**

Cryptococcal meningitis accounts for 15% of AIDS-related mortality. Cryptococcal antigen (CrAg) is detected in blood weeks before onset of meningitis, and CrAg positivity is an independent predictor of meningitis and death. CrAg screening for patients with advanced HIV and preemptive treatment is recommended by the World Health Organization, though implementation remains limited. Our objective was to evaluate costs and mortality reduction (lives saved) from a national CrAg screening program across Uganda.

**Methods:**

We created a decision analytic model to evaluate CrAg screening. CrAg screening was considered for those with a CD4<100 cells/μL per national and international guidelines, and in the context of a national HIV test-and-treat program where CD4 testing was not available. Costs (2016 USD) were estimated for screening, preemptive therapy, hospitalization, and maintenance therapy. Parameter assumptions were based on large prospective CrAg screening studies in Uganda, and clinical trials from sub Saharan Africa. CrAg positive (CrAg+) persons could be: (a) asymptomatic and thus eligible for preemptive treatment with fluconazole; or (b) symptomatic with meningitis with hospitalization.

**Results:**

In the base case model for 1 million persons with a CD4 test annually, 128,000 with a CD4<100 cells/μL were screened, and 8,233 were asymptomatic CrAg+ and received preemptive therapy. Compared to no screening and treatment, CrAg screening and treatment in the base case cost $3,356,724 compared to doing nothing, and saved 7,320 lives, for a cost of $459 per life saved, with the $3.3 million in cost savings derived from fewer patients developing fulminant meningitis. In the scenario of a national HIV test-and-treat program, of 1 million HIV-infected persons, 800,000 persons were screened, of whom 640,000 returned to clinic, and 8,233 were incident CrAg positive (CrAg prevalence 1.4%). The total cost of a CrAg screening and treatment program was $4.16 million dollars, with 2,180 known deaths. Conversely, without CrAg screening, the cost of treating meningitis was $3.09 million dollars with 3,806 deaths. Thus, despite the very low CrAg prevalence of 1.4% in the general HIV-infected population, and inadequate retention-in-care, CrAg screening averted 43% of deaths from cryptococcal meningitis at a cost of $662 per death averted.

**Conclusion:**

CrAg screening and treatment programs are cost-saving and lifesaving, assuming preemptive treatment is 77% effective in preventing death, and could be adopted and implemented by ministries of health to reduce mortality in those with advanced HIV disease. Even within HIV test-and-treat programs where CD4 testing is not performed, and CrAg prevalence is only 1.4%, CrAg screening is cost-effective.

## 1. Introduction

Cryptococcal meningitis remains a leading cause of death in persons with advanced HIV infection, accounting for 15% of AIDS-related mortality. [1] Mortality from meningitis remains high at 50 to 70% in sub-Sahara Africa, due to delays in presentation to care, need for complex management requiring serial lumbar punctures, and limited access to optimal antifungal medications. [2–4] Cryptococcal antigen (CrAg) can be detected in the blood weeks before onset of meningitis, and CrAg positivity is an independent predictor of meningitis and death.[5–7] Screening for CrAg amongst those with advanced HIV, and preemptively treating those CrAg positive with fluconazole has been evaluated in a randomized controlled trial in Zambia and Tanzania, and, alongside adherence counseling, demonstrated a 28% reduction in mortality[8]. CrAg screening and preemptive treatment for patients with advanced HIV is now recommended by the World Health Organization (WHO) and numerous national HIV programs.[9]

Ugandan national HIV consolidated guidelines recommend CrAg screening in HIV-infected persons with a CD4 cell count ≤100 cells/μL, although implementation remains limited. Data from the Ugandan national CrAg register suggest that in the first quarter of 2017 only 19% of those eligible for CrAg screening were actually screened. Of these, only 65% of CrAg-positive patients received fluconazole preemptive therapy. A clear disparity remains between national guidelines and actual implementation. As ministries of health attempt to roll out CrAg screening and treatment on a broader scale, estimated costs of screening programs and the implications for meningitis treatment and hospitalization costs are lacking. Analysis of costs of screening and possible mortality reductions at varying levels of national implementation are needed to identify opportunities to improve upon the current standard of little to no CrAg screening.

Based on a detailed analysis of Ugandan costs for CrAg screening and meningitis treatment from the perspective of the Ugandan Ministry of Health, the first objective of this study was to evaluate costs and mortality reductions (lives saved) of improved implementation of the national CrAg screening program across Uganda. The analysis assumed a lab-based reflex screening strategy, where a CrAg test is completed for all patients with a CD4 count less than 100 cells/μL per current Ugandan HIV treatment guidelines recommendations. A second objective was to consider how the basic model results could change with alternative meningitis treatment regimens, some of which involve medications that are not yet available in Uganda (e.g., flucytosine). The third objective was to extend the analysis to integrating CrAg screening into HIV test-and-treat programs that initiate antiretroviral therapy (ART) immediately in those newly diagnosed with HIV infection in the absence of prior CD4 testing. This universal ART initiation strategy is also now recommended by the WHO [10], and CrAg screening programs will need to adapt in locations where ART initiation occurs in the absence of a baseline CD4 result at the time of initiation.

## 2. Methods

A decision analytic model was developed to evaluate CrAg screening and treatment as two separate stages: 1) screening for CrAg; and 2) treatment for CrAg-positive persons, which includes preemptive treatment of those asymptomatic CrAg-positive, as well as hospitalization and treatment for meningitis for those symptomatic CrAg-positive, and those missed by the screening program. Each stage is summarized below. The general model and analysis follows previous analyses from South Africa.[11,12] The details of the model structure, parameters, and cost details have been adapted to apply to Uganda.

### 2.A. CrAg screening for those with CD4≤100 cells/μL

**Fig 1:**
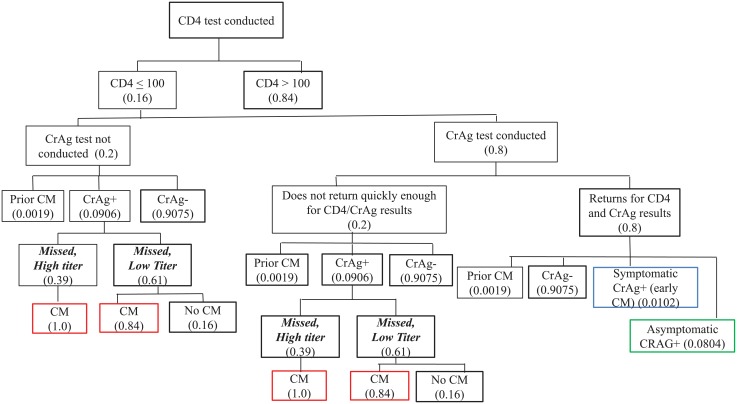
Base case model of CrAg screening reflexively for CD4 counts <100 cells/μL.

The CrAg screening stage identified CrAg-positive individuals in the HIV-infected population with a CD4 cell count ≤100 cells/μL, and potential CrAg-positive persons who were missed by the screening program—either because a CrAg test was not completed after their CD4 test or they were tested for CrAg but did not return for their lab results (Fig 1). Table 1 describes all input parameters and sources of data for CrAg screening population. The model results presented as the “base case” use the parameter assumptions in Table 1. See S1 Materials for further details regarding input parameters.

**Table 1:** Description of HIV-infected population screened in CD4 testing and reflexive lab screening model.

Population screened	Probability	Source
**CD4 count ≤100 cells/μL**	0.16	[21, 22]
**CD4≤100 cells/μL CrAg screened**	0.8	Assumption
**Return to clinic for CrAg results**	0.8	Assumption
**Asymptomatic CrAg+ receives preemptive treatment**	1.0	
**CrAg prevalence**		
** CrAg negative**	0.9075	[14]
** Prior CCM**	0.0019	[14]
** Incident CrAg +**	0.0906	[14]
** Symptomatic CrAg+**	0.0102	[14]
** Asymptomatic CrAg+**	0.0804	[14]
** Of incident asymptomatic CrAg+**		
** High titer**	0.39	[14]
** Low titer**	0.61	[14]
**CrAg+ outcomes**		
** CrAg+ high titer, no preemptive fluconazole or ART, develops CM**	1.0	[19]
** CrAg+ low titer, no preemptive fluconazole or ART, subsequently develops CM**	0.50	[6, 23]
** CrAg+ high titer, receives preemptive fluconazole, subsequently survives**	0.64	[14]
** CrAg+ low titer, receives preemptive fluconazole, survives**	0.86	[14]
** CrAg+ symptomatic presents to hospital**	0.73	[15]
** CrAg+ fails fluconazole, presents to hospital**	0.80	Assumption
**Symptomatic Meningitis outcomes**		
** CM who present to hospital**	0.80	Assumption

In Fig 1, for those with a CD4 count ≤100 cells/μL, it was assumed that 80% were CrAg screened and 80% of those tested returned for their CD4 and CrAg results (CrAg negative or CrAg positive). CrAg positive persons were disaggregated into three categories: (1) asymptomatic and thus eligible for preemptive treatment with fluconazole; (2) symptomatic with meningitis who should be hospitalized; and (3) CrAg-positive due to a history of cryptococcal infection. Those with a history of cryptococcal infection remain CrAg positive for a prolonged period, and this lab result was considered an artifact, as they would not benefit from further antifungal treatment.

Those who were not tested for CrAg, or for those who had a CrAg test performed but did not return for results, were also classified as CrAg negative or CrAg positive as described above. These categories were used to stratify risk of meningitis and death. CrAg testing was presumed to occur using the lateral flow assay (LFA) (Immy, Norman OK), which was >99% sensitive and specific.

**Fig 2:**
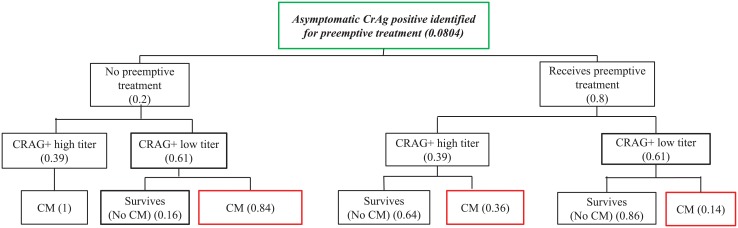
Base case model of treatment of Asymptomatic CrAg positive persons.

Asymptomatic CrAg positive persons were further categorized as having a high titer (≥1:160), and therefore more likely to progress to meningitis and/or death, or as having a low titer (<1:160), and therefore less likely to develop meningitis or death (Fig 2).[2,13] Prevalence of high and low titer status was taken from the largest CrAg screening study performed in Uganda.[14] Titer was not known to clinicians and did not affect clinical care.

### 2.B. Preemptive treatment for asymptomatic CrAg positive persons

For asymptomatic CrAg positive persons, we assumed that all who returned for results were eligible for preemptive treatment. It was assumed that for asymptomatic CrAg positive persons with a high titer who did not receive preemptive fluconazole, all progressed to meningitis (Fig 2). Those with a low titer had a lower probability of progression to meningitis (See S1 Materials).

### 2.C. Treatment for cryptococcal meningitis

**Fig 3:**
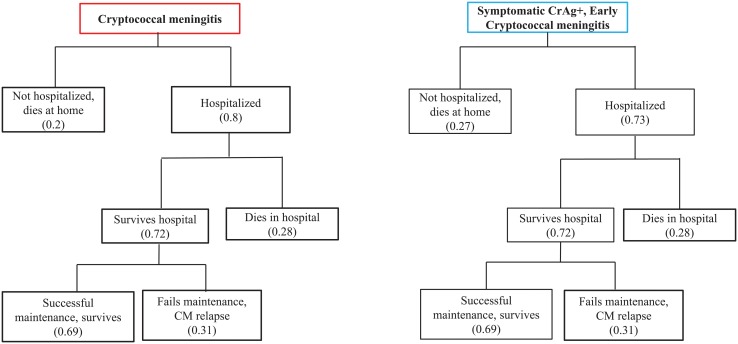
Left: Base case model of care of those identified with cryptococcal meningitis after not being screened, not returning to clinic, not receiving preemptive treatment, or failing preemptive treatment. Right: Base case model of care of those identified as symptomatic blood CrAg positive, with early cryptococcal meningitis.

We assumed that for those with meningitis (whether at the time of screening, or not screened, or did not return for results, or did not receive preemptive treatment, or who failed preemptive treatment), 0.2 never presented to a hospital for treatment and 0.8 presented to a hospital for treatment. (Fig 3). This was an estimate, as no studies have evaluated what proportion of Ugandan patients with meningitis present to medical care. Symptomatic CrAg positive persons (with early meningitis) who returned for their CrAg results, had a 0.73 probability of presenting to the hospital for treatment for meningitis. [15]

In the base case model, those hospitalized for cryptococcal meningitis were treated with amphotericin + fluconazole for 14 days, per the standard of care in Uganda. See S1 Materials for further details regarding 2-week survival and 1-year survival estimates.

### 2.D. Health outcomes

Patients were assumed to have three primary outcomes: survived, died, or unknown. The unknown were a small number of persons who developed meningitis, survived hospitalization but then developed recurrent meningitis during their post-hospital maintenance treatment phase. These patients either died without returning to a hospital or presented back to a hospital for further care.

While deaths avoided (lives saved) was used as the main health outcome, this outcome could be translated into disability adjusted life years (DALYs) avoided if needed. For example, for persons who died from cryptococcal meningitis, the base case assumption could be made that the average age of death is 30 to 34 years. In general, average life expectancy for this age group is an additional 31 years [16]. With a 3% discount rate, and 31 years of life lost from a death, 20.6 DALYs were lost per death (all from years of life lost), so 20.6 DALYs were avoided per death prevented.

### 2.E. Screening and treatment costs

For the base case results, the screening and treatment costs were based on the information in Table 2. All costs were reported in 2016 US dollars, and assumed to have been borne fully by the Ugandan ministry of health. The 2016 annual average exchange rate was 3,420 Ugandan shillings (UGX) for 1 US dollar [17]. CrAg testing was estimated at $3.41 per test, based on the cost of the lateral flow assay, import costs per the manufacturer, shipping, and labor.

**Table 2:** Input costs for CrAg screening and treatment.

	unit	USD	Notes
**CrAg test cost**		$3.41	
	CrAg LFA	$2.00	Source–Immy (personal communication)
	Import cost	$0.80	Source–Immy (personal communication)
	Shipping	$0.03	0.03 per test to ship 20,000 tests
	Labor	$0.58	Lab worker salary for 10 minutes to perform test
**Fluconazole**	200mg tablet	$0.14	[18]
**Preemptive fluconazole course**		$39.06	Including 6 months maintenance on 200mg daily
**Hospitalization**			
** Hospital stay**	$9.94 per day x 14 days	$139.18	
** Lab testing**		$89.18	1 CBC, 3 Cr, 3 K, 3Na, CSF analysis, CSF culture, CrAg
** Supplies**	$3.41 per day x 14 days	$47.69	
** LPs**	$9.29 per LP x 3	$27.88	
** Personnel**	$6.64 per day x 14 days	$93.03	Nurse, Doctor, HIV counselor, Phlebotomist, Lab tech
** Amphotericin**	50mg per day x 14 days	$165.94	[18]
**Hospitalization total**		**$562.90**	
**Post hospitalization consolidation and maintenance with fluconazole**		$66.78	1 year

Fluconazole costs of $0.14 per 200mg tablet were based on information from the Joint Medical Store (JMS), a major Ugandan national supplier.[18] Thus, the full course of preemptive treatment with fluconazole, including 2 weeks at 800mg daily, followed by 10 weeks at 400mg daily, and an additional 3 months maintenance therapy at 200mg was $39.06.

Hospitalization costs for cryptococcal meningitis were further described in the S1 Materials. Including post-hospitalization consolidation and maintenance fluconazole therapy for 1 year, the total cost of hospitalization and therapy for a person with cryptococcal meningitis in Uganda was estimated at $630.

### 2.F. Sensitivity analyses with existing recommended drug regimens

Sensitivity analyses (SA) were first conducted to explore the impacts on costs and health outcomes for improved implementation of CrAg screening, preemptive treatment, and meningitis treatment given currently available drug regimens. Compared to the base case analysis outlined above, we first investigated four main alternatives:

SA.a No screening, no preemptive treatment, no hospitalization for meningitis, only deaths from meningitis;
SA.b No screening, no preemptive treatment, 50% of those with meningitis were hospitalized;
SA.c No screening, no preemptive treatment, 80% of those with meningitis were hospitalized;
SA.d CrAg screening with preemptive treatment, 50% of those with meningitis were hospitalized;

Reduced proportion of patients with meningitis hospitalized for treatment at 50% is more realistic in rural areas.

### 2.G. Sensitivity analyses with alternative drug regimens

An additional set of sensitivity analyses is presented to explore the impacts of alternative meningitis treatment drug regimens on overall costs and health outcomes. We evaluated three alternate meningitis treatment strategies summarized in Table 3. See S1 Materials for further details on input parameters.

**Table 3:** Hospital costs and survival with alternative drug regimens.

Regimen	Duration of induction treatment	Duration of hospital admission	Cost of hospitalization	Probability of 2-week survival*	Probability of 1-year survival	Notes
**Amphotericin 1mg/kg daily + fluconazole 1200mg (base case)**	14 days	14 days	$562.90	0.82*	0.45	
**Fluconazole 1200mg**	14 days	7 days	$295.81	0.3	0.45	No lab monitoring
**Amphotericin 1mg/kg daily + flucytosine 50mg/kg**	7 days	7 days	$557.65	0.88*	0.58	[25]
**Fluconazole 1200mg daily + Flucytosine 50mg/kg**	14 days	7 days	$510.83	0.85*	0.51	No lab monitoring [25]

* 2-week survival taken from ACTA trial. For the model, 0.1 was subtracted from 2-week survival reported above to reflect real-world survival which is worse than the clinical trial setting.

### 2.H. CrAg screening in a HIV test-and-treat program (without baseline CD4 count)

**Fig 4:**
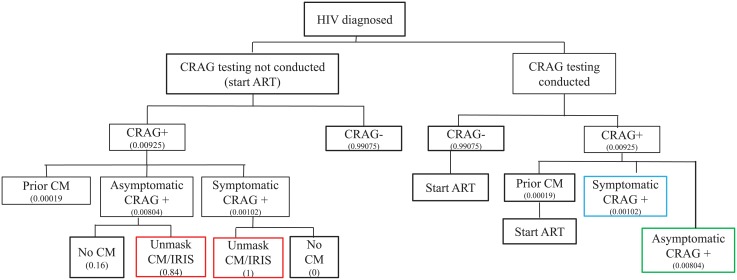
CrAg screening within HIV test-and-treat model.

**Fig 5:**
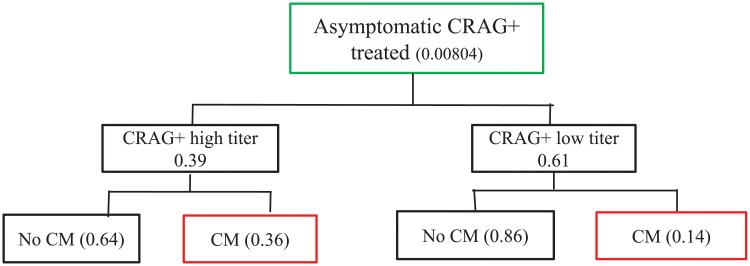
Treatment of Asymptomatic CrAg positive persons within the HIV test & treat model.

We also modeled one strategy for implementing CrAg screening into the context of HIV test-and-treat programs that initiate patients without a baseline CD4 cell count (Figs 4 and 5). Here, we considered that all those diagnosed with HIV infection were CrAg screened before a baseline CD4 count is known. All parameter assumptions for this variation of the model are also reported in Table 4. Further details regarding input parameters are described in the Supplemental materials (S1 Materials). In this model, there is a risk of unmasking cryptococcal meningitis for those CrAg+ persons who start ART without antifungal therapy.

**Table 4:** Description of HIV-infected Population Screened in HIV test & treat model.

Population screened	Probability	Source
**CD4 count ≤100 cells/μL**	0.16	[21, 22]
**CD4≤100 cells/μL CrAg screened**	0.8	Assumption
**Return to clinic for CrAg results**	0.8	Assumption
**Asymptomatic CrAg+ receives preemptive treatment**	1.0	
**CrAg prevalence**		
** CrAg negative**	0.9075	[14]
** Prior CCM**	0.0019	[14]
** Incident CrAg +**	0.0906	[14]
** Symptomatic CrAg+**	0.0102	[14]
** Asymptomatic CrAg+**	0.0804	[14]
** Of incident asymptomatic CrAg+**		
** High titer**	0.39	[14]
** Low titer**	0.61	[14]
**CrAg+ outcomes**		
** CrAg+ high titer, no preemptive fluconazole or ART, develops CM**	1.0	[19]
** CrAg+ low titer, no preemptive fluconazole or ART, subsequently develops CM**	0.50	[6, 23]
** CrAg+ high titer, receives preemptive fluconazole, subsequently survives**	0.64	[14]
** CrAg+ low titer, receives preemptive fluconazole, survives**	0.86	[14]
** CrAg+ symptomatic presents to hospital**	0.73	[15]
** CrAg+ fails fluconazole, presents to hospital**	0.80	Assumption
**Symptomatic Meningitis outcomes**		
** CM who present to hospital**	0.80	Assumption
** Unmasking CM on ART, survives hospitalization with treatment**	0.50	[24]

## 3. Results

### 3.A. CD4 testing and reflexive CrAg screening

In the absence of any treatment for cryptococcal meningitis, an estimated 10,075 patients would die annually (Table 5). By comparison, in the base case model analysis using the assumptions in Tables 1 and 2, 128,000 patients are screened for a cost of $436,314. Of these, 8,233 asymptomatic CrAg positive patients receive preemptive treatment for a cost of $295,431. A total of 4,671 patients develop meningitis, and 4,363 are hospitalized for a total treatment cost of $2.62 million dollars. Compared to no screening and no treatment (SA.a Table 5), CrAg screening and treatment in the base case costs $3,356,724 compared to doing nothing, and saves 7,320 lives, for a cost of $459 per life saved.

**Table 5:** Sensitivity of results to level of CrAg screening and proportion of patients with meningitis treated.

Results	Strategy	Cost of Screening	Cost of preemptive treatment	Cost of treatment for meningitis	Total Cost	Deaths	Note
SA.a	**No screening or treatment for CM**	0	0	0	0	10075	Do nothing
SA.b	**No screening, 50% hospitalized (CM treatment with amphotericin + fluconazole)**	0	0	$2,528,052	$2,528,052	6448	Dominated by SA.d if slightly larger budget possible
SA.c	**No screening, 80% hospitalized. CM treatment with amphotericin + fluconazole**	0	0	$4,044,882	$4,044,882	4272	Dominated by SA.d and base case
SA.d	**CrAg screening, 50% hospitalized (CM treatment with amphotericin + fluconazole)**	$436,314	$295,431	$2,078,920	$2,810,665	3538	CrAg screening with 50% of patients being hospitalized has essentially the same total costs as the same amount of hospitalization without screening (SA.b), but an additional 2,910 lives are saved annually.
Base case results	**CrAg screening, 80% hospitalized (CM treatment with amphotericin + fluconazole)**	**$436,314**	**$295,431**	**$2,624,979**	**$3,356,724**	**2755**	

The results for cost and lives saved for the alternative scenarios (SA.b—SA.d) are also reported in Table 5. For example, comparing SA.d to SA.b, CrAg screening with 50% of patients being hospitalized has essentially the same total costs as the same amount of hospitalization without screening (SA.b), but saves an additional 2,910 lives due mainly to preemptive treatment and earlier identification of meningitis patients (symptomatic CrAg+ identified).

**Fig 6:**
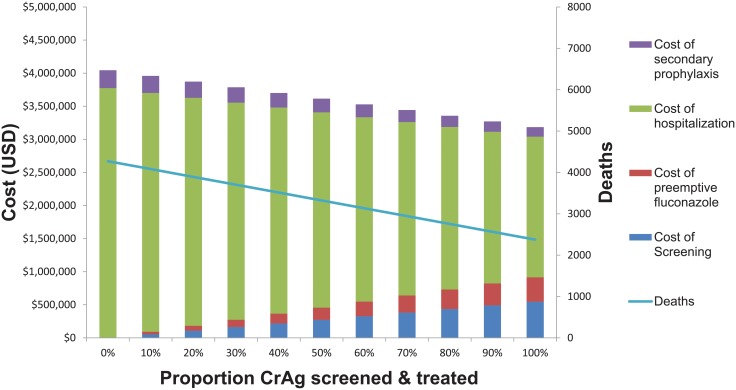
Cost of CrAg screening and preemptive treatment with differential levels of implementation. With 100% CrAg screening and treatment, 1900 lives are saved (44%) and $860,000 dollars, compared to no screening. Even with a small increase in CrAg screening from 0 to 10%, there are cost savings and deaths averted.

As an extension of the sensitivity analyses reported in Table 5, Fig 6 summarizes costs and health outcomes (total deaths) with various levels of implementation of CrAg screening and treatment. All assumptions are kept constant, as in Fig 1, Tables 1 and 2: those hospitalized for meningitis are treated with amphotericin + fluconazole, but we varied the proportion CrAg screened from 0 to 1. All those screened are assumed to have returned to clinic and receive preemptive treatment (whereas in the base case analysis it is assumed that 80% of those eligible are screened, and only 80% return to clinic for results; all those who return to clinic are treated). Fig 6 illustrates how changing the proportion of screened persons affects deaths and costs. CrAg screening for all those eligible with a CD4 cell count <100 cells/μL, compared to no screening, saves over $860,000 dollars and save 1,900 lives (39,078 DALYs), at $453 saved per death averted.

### 3.B. Alternative treatment regimens

When evaluating alternative treatment regimens, all parameters are kept the same as in Fig 1 and in Tables 1 and 2, but hospitalization costs and survival are varied in accordance with previous published outcomes. Hospital costs and survival parameters for alternative meningitis treatment regimens are summarized in Table 3. Total costs and survival with each treatment strategy are summarized in Table 6. While fluconazole alone for meningitis treatment in the absence of CrAg screening saves lives compared to doing nothing (at a cost of $633 per life saved), including CrAg screening with this drug regimen costs less than $200,000 additionally, and saves 2,700 more lives. Notably, in the absence of a CrAg screening program, flucytosine-based regimens do not improve survival compared to CrAg screening and meningitis treatment with amphotericin + fluconazole. Compared to the base case regimen of amphotericin + fluconazole for treatment of cryptococcal meningitis, additional lives can be saved with the use of a flucytosine-based regimen for cryptococcal meningitis with concurrent CrAg screening.

**Table 6:** Sensitivity of results to alternative meningitis treatment strategies.

	Strategy	Cost of Screening	Cost of preemptive treatment	Cost of meningitis treatment	Total Cost	Deaths	Comments
SA.a	0. No screening or treatment for CM	0	0	0	0	10075	
Other Regimen	1. No screening, CM treatment with fluconazole only	0	0	$1,529,751	$1,529,751	7657	
Other Regimen	2. CrAg screening, CM treatment with fluconazole only	$436,314	$295,431	$970,163	$1,701,908	4957	Screening with modestly effective drug saves 2700 more lives compared to no screening (strategy 2 compared to 1)
Other Regimen	3. No screening, CM treatment with fluconazole + flucytosine	0	0	$3,806,382	$3,806,382	4030	Total costs with a very effective drug for treatment without screening is significantly higher than screening with a less effective drug (strategy 3 compared to 2). Dominated by base case results.
Other Regimen	4. No screening, CM treatment with amphotericin + flucytosine	0	0	$4,247,002	$4,247,002	3788	Similar comment as above. Dominated by base case results.
**Base case results**	**CrAg screening, CM treatment with amphotericin + fluconazole**	**$436,314**	**$295,431**	**$2,624,979**	**$3,356,724**	**2755**	
Other Regimen	5. CrAg screening, CM treatment with fluconazole + flucytosine	$436,314	$295,431	$2,468,561	$3,200,306	2597	Based on current cost assumptions, access to flucytosine would have modest additional improvements on lives saved with essentially similar total costs.
Other Regimen	6. CrAg screening, CM treatment with amphotericin + flucytosine	$436,314	$295,431	$2,753,776	$3,485,521	2440	Same comment as above.

**Fig 7:**
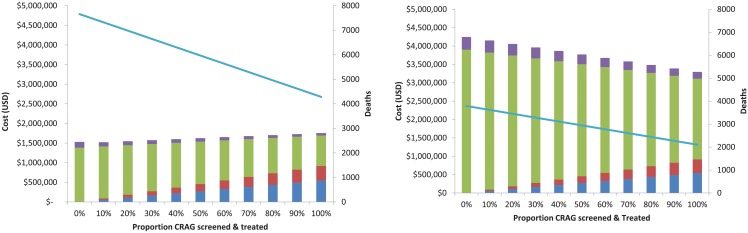
Cost savings of CrAg screening with different treatment regimens for those with cryptococcal meningitis. Left: CrAg screening and preemptive treatment where cryptococcal meningitis is treated with fluconazole alone. Right: Cryptococcal meningitis is treated with amphotericin + flucytosine. For analyses where meningitis was treated with fluconazole alone (which is highly ineffective), CrAg screening is cost-effective, but not cost-saving, because the treatment for meningitis is inexpensive. However, full implementation of CrAg screening resulted in a 44% reduction in mortality, given the poor efficacy of fluconazole in the treatment of meningitis. Conversely, if meningitis is treated with amphotericin + flucytosine, CrAg screening is cost-saving because treatment for meningitis is expensive, and reduces mortality. Mortality reductions are less dramatic with meningitis regimens that are more effective. In all scenarios, CrAg screening reduced mortality and was cost-effective.

Fig 7 depicts costs and deaths with differential levels of CrAg screening using two different treatments for treatment of cryptococcal meningitis: a) fluconazole alone, and b) amphotericin + flucytosine. Unlike the base case scenario which assumes 80% of eligible persons are screened, and 80% return to clinic, here we vary the proportion screened from 0% to 100%, and assume that all those screened return to clinic and are treated. This allows us to explore varying survival outcomes and costs related to different levels of implementation of the CrAg screening and preemptive treatment intervention.

Fig 7 illustrates that with the use of fluconazole alone for treatment of meningitis, addition of a CrAg screening program costs approximately $200,000. However, the relative ineffectiveness of this agent in treating meningitis results in substantial mortality. CrAg screening reduces these deaths by 3,375. Conversely, survival from meningitis is higher when amphotericin + flucytosine are used for treatment; in this setting, CrAg screening reduces deaths by 1,685, but the cost-savings of implementing a CrAg screening program are more evident, because of the high cost of amphotericin + flucytosine.

If meningitis is treated with amphotericin + flucytosine or fluconazole + flucytosine, CrAg screening is cost-saving, and reduces mortality. CrAg screening results in lower costs and fewer deaths compared to no screening. In the absence of flucytosine, CrAg screening and treatment of breakthrough meningitis with amphotericin and fluconazole averts the most deaths.

### 3.C. HIV test-and-treat model

In the context of HIV test-and-treat programs, we assume that 1 million HIV cases enter care per year. In the base case HIV test-and-treat model, using the assumptions in Table 4, 1 million HIV-infected persons, 800,000 are screened, and 640,000 of those screened return for results. Of these, 8,233 are incident CrAg positive infections.

Screening costs $2.18 million and preemptive treatment for those 8233 asymptomatic CrAg-positive identified for treatment costs $295,431. Hospitalization for the 3,540 persons with breakthrough meningitis costs $1,586,092 dollars—assuming these patients were treated with amphotericin and fluconazole for 14 days, per standard of care. In this model, the cost of CrAg screening is 52% of the entire cost of screening and treatment, because this model assumes a much lower prevalence of CrAg positivity (1.4%). In the prior model, based on reflexive CD4 testing, CrAg screening costs comprise 19% of screening and treatment costs.

The total cost of a CrAg screening and treatment program is $4.16 million, with 2,180 known deaths. Conversely, without CrAg screening, the cost of treating meningitis is $3.09 million with 3,806 deaths. Thus, despite the very low CrAg prevalence in the HIV test-and-treat model, CrAg screening averts 43% of deaths, at a cost of $662 per death averted.

**Fig 8:**
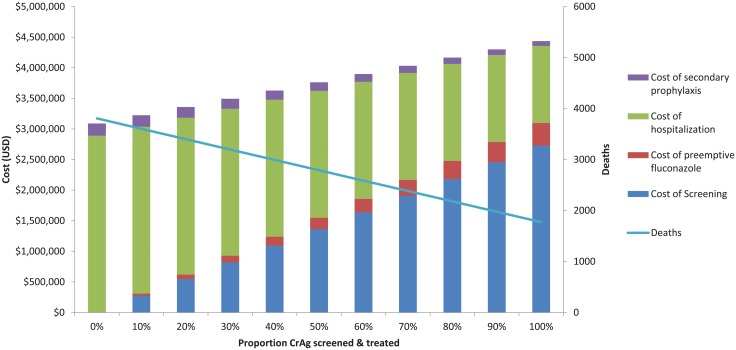
Cost of CrAg screening and preemptive treatment with differential levels of implementation with an HIV test-and-treat model. With 100% CrAg screening and treatment, 53% of deaths are averted at a cost of $662 per death averted.

Fig 8 illustrates a variation of the base case analysis–deaths and costs with different levels of CrAg screening. Here, we assume the same parameters outlined in Table 4, but we assume that all those who are screened return to clinic and receive preemptive treatment. Without any CrAg screening in a Ugandan HIV test-and-treat program, the ministry of health can anticipate spending $3.09 million dollars for hospitalization for meningitis, and expect 3,806 deaths. Conversely with full implementation of a CrAg screening and preemptive treatment program, costs would be $4.43 million, with 1,773 deaths. Thus, a national CrAg screening program with perfect implementation would avert 53% of deaths, at a cost of $662 per death averted.

## 4. Discussion

In our analysis, implementing CrAg screening generally reduced total costs and saves lives given high hospitalization costs and mortality related to meningitis. Our analysis highlights that the treatment regimen employed for cryptococcal meningitis and its cost are integral to estimating/calculating the cost-benefit of CrAg screening. CrAg screening will predominantly save money to the healthcare system when an expensive but highly effective regimen such as amphotericin + flucytosine is used to treat cryptococcal meningitis. In contrast, when fluconazole alone, a low-cost but far less effective agent is used to treat cryptococcal meningitis (as is common in rural settings), CrAg screening predominantly averts deaths. In all scenarios, regardless of treatment regimen or proportion hospitalized for meningitis, CrAg screening saves lives and money, assuming that treatment is 77% effective in averting mortality.

Importantly, compared to the base case scenario of CrAg screening and meningitis treatment with amphotericin + fluconazole, flucytosine-based meningitis treatment regimens only save more lives if implemented in the context of a CrAg screening and preemptive treatment program. Flucytosine-based regimens for the treatment of meningitis in the absence of CrAg screening and treatment do not save more lives than current standard of care (amphotericin + fluconazole) in conjunction with CrAg screening and treatment.

The benefits of CrAg screening carry over to a HIV test-and-treat environment even when CrAg prevalence is 1.4% or lower. Despite this very low CrAg prevalence among all newly diagnosed HIV patients, CrAg screening all HIV-infected persons, when no CD4 count is available, averts 53% of deaths and at a cost of $662 per death averted. The concept of CrAg screening within HIV test-and-treat programs is novel and valuable, given the WHO recommendation to expedite treatment in those newly HIV diagnosed. CrAg screening programs can confidently adapt to CrAg screen all persons entering care in test-and-treat programs in which CD4 testing is not being performed. With benefit at such a low prevalence, consideration for CrAg screening in other contexts–such as reflexively in those with virologic failure, or in those with CD4 between 100 and 150 cells/μL could be further investigated. In the context of HIV test-and-treat programs in Uganda, in the absence of CD4 testing, national guidelines currently recommend cryptococcal testing if a person has signs or symptoms of meningitis. Thus, CrAg testing is not being used as a screening tool for asymptomatic persons, but to detect meningitis in those who are already symptomatic. In our prior cohort, we found that those who were symptomatic at time of CrAg testing had 54% mortality, with median time to death of 9 days.[19]

Ministries of health could invest in national CrAg screening programs, knowing that the cost of screening is offset by the averted deaths and associated costs from cryptococcal meningitis, regardless of meningitis treatment strategy. Investment in such a screening program requires a steady supply of CrAg tests and fluconazole for preemptive treatment. In Uganda we note dependence on the Pfizer Diflucan Partnership Program, but this arrangement has not resulted in the availability of fluconazole in many parts of the country. Thus, fluconazole stock outs are widespread. Investment in an adequate supply of fluconazole, a drug that is cheap and should be accessible, will save costs and lives. Conversely if no action is taken, Ministries of health will spend more in hospitalization costs to treat cryptococcal meningitis, with increased mortality.

The principal limitations of our analysis are related to input parameters of our model. We used data from large cohort studies from Uganda and clinical trials from sub Saharan Africa, but some parameters are unknown. For those unknown inputs, estimates were based on Ugandan cryptococcal studies. We expect that estimates will vary from country to country (for example, South Africa has much higher hospitalization costs), and even within a country, depending on public or private setting. Thus, there is likely variability in our estimates when applied to other settings. However, in all of our sensitivity analyses, regardless of the posited proportion hospitalized, cryptococcal treatment regimen, public or private hospitalization location, CrAg screening is always favorable.

Our base case analyses assumed only 80% of eligible persons were CrAg screened, and 80% of those CrAg+ returned to clinic for treatment. Thus, our conclusions are conservative, in an attempt to understand real-world limitations in implementing a national screening program. We did not account for those with contraindications to fluconazole such as pregnant women, because there is no consensus or guideline as to how best to treat these women.

Our model draws attention to important areas for future research. Namely, it is unknown whether CrAg-positive persons recently started on ART progress to meningitis or death at rates similar to those who are ART-naïve. Prospectively collected data on CrAg prevalence in HIV test-and-treat programs will be valuable to further cost-benefit analyses. The risk of meningitis and/or death amongst CrAg positive persons in test-and-treat programs who receive ART but no preemptive fluconazole has not been studied. This likely varies by CD4 count and CrAg titer. Timing of ART initiation in CrAg-positive persons receiving fluconazole has not been studied. The Southern African HIV Clinician’s Society recommends delaying ART for 2 weeks based on evidence from cryptococcal meningitis.[20] However, if there is no benefit to delaying ART in this population, initiating preemptive fluconazole and ART together would likely reduce loss to follow up. Finally, the efficacy of 6 months of maintenance fluconazole therapy in asymptomatic CrAg positive persons is unclear. If proved unnecessary, CrAg screening programs would be even less costly—although the cost of preemptive fluconazole constitutes a small portion of total costs.

As HIV programs shift from CD4 stratification to a test-and-treat model, the management of persons with advanced HIV disease has not been clearly defined. While the majority may benefit from expedited initiation of ART, those with advanced disease at risk for cryptococcal infection and tuberculosis may require further consideration of opportunistic infections before ART initiation. Our analysis demonstrates that CrAg screening of all persons entering HIV care, ideally with a point-of-care assay, initiating ART in those who test CrAg-negative, and preemptively treating those testing CrAg positive with fluconazole is cost-effective and lifesaving.

## Supporting information

S1 Materials Supplemental materials contains methods and input parameters for sensitivity analyses. (DOCX)

S1 Model Uganda CrAg Model contains the working base case model used. (XLSX)
