# Supplementary material for: Evaluation of a national cryptococcal antigen screening program for HIV-infected patients in Uganda: A cost-effectiveness modeling analysis
Source: PLoS One. 2019 Jan 10;14(1):e0210105. doi: 10.1371/journal.pone.0210105 (PMC6328136; doi:10.1371/journal.pone.0210105)
Supplement: S1 Materials — (DOCX) [file pone.0210105.s001.docx]

**Supplemental Methods**

**Supplemental 2.A. CrAg screening for those with CD4<100 cells/µL**

Based on Ugandan Ministry of Health data, we assumed 16% of all CD4 tests are <100 cells/µL, which includes both those ART-naïve and those already ART-experienced. CrAg prevalence was taken from the largest prospective CrAg screening study in Uganda.(1)

**Supplemental 2.B. Preemptive treatment for asymptomatic CrAg positive persons**

Those with low CrAg titers who receive preemptive fluconazole have a 0.86 probability of survival, compared to 0.5 probability of survival without preemptive treatment. Similarly, those with high CrAg titers who receive preemptive fluconazole have a 0.64 probability of survival, compared to 0 probability of survival without preemptive therapy. The base model assumptions are taken from the largest Ugandan cohort study of outcomes of CrAg positive persons who received treatment.(1)

**Supplemental 2.C. Treatment for cryptococcal meningitis**

In the base case model, those hospitalized for cryptococcal meningitis are treated with amphotericin + fluconazole requiring a 14 day hospitalization, per the standard of care in Uganda. There is a 0.72 probability of surviving the hospitalization, and a 0.57 probability of surviving at 10 weeks. These survival figures are taken from the published by the ACTA trial, which evaluated cryptococcal meningitis treatment outcomes in sub Saharan Africa.(2) In the base case model, we subtracted 2-week survival from this clinical trial by 10% to reflect probable outcomes in routine care. Those that do not adhere to maintenance antifungal therapy develop meningitis relapse and/or die. Survival post hospitalization was taken from 10-week survival published by the ACTA trial.(2) Ten-week survival was multiplied by 0.79 to estimate 1-year survival after cryptococcal meningitis.(3) Thus 1-year survival for cryptococcal meningitis in Uganda, after being treated with amphotericin and fluconazole was estimated at 0.45, which correlates with published survival estimates.(3)

**Supplemental 2.E. Screening and Treatment Costs**

Hospitalization costs for cryptococcal meningitis includes room and board costs for a hospital stay in a public facility of 14 days, lab testing and monitoring, hospital supplies, lumbar punctures (1 diagnostic, and 2 therapeutic), medical personnel, and medication costs. Lab testing includes initial diagnosis of meningitis (CSF CrAg, CSF culture, CSF analysis), but also monitoring for toxicity while on amphotericin with complete blood counts, renal function, and electrolytes. Hospital supplies included IV tubing, IV fluids, gauze, bandages etc. Lumbar puncture costs include a manometer, but also CSF collection tubes, and LP needles. Personnel costs include nurses, doctors, HIV counselors, phlebotomists, and laboratory technician. Monthly salaries were obtained from the Mulago national referral hospital –a public tertiary hospital in Uganda. From this, daily salaries were calculated. It was assumed that each staff saw 20 patients per day, so a per patient daily salary was calculated, and then multiplied by 14, to calculate the cost of each hospital worker for a patient hospitalized for 2 weeks. Medication costs were taken from the published JMS costs from the Management Sciences for Health (MSH) website.(4)

**Supplemental 2.G. Sensitivity analyses with alternative drug regimens**

Ten-week survival as measured in the ACTA trial, was multiplied by 0.79 to yield estimates of 1-year survival. These strategies include total costs and total deaths with each strategy (summarized in Table 3) using all the other parameter assumptions in Table 1 and Table 2. Though flucytosine is not currently available in Uganda, efforts to improve access to this crucial drug are underway, and flucytosine is the recommended first-line treatment for cryptococcal meningitis.(5)

**Supplemental 2.H. CrAg screening in a HIV test-and-treat program (without baseline CD4 count)**

We assume that 16% of all new HIV diagnoses would have a CD4<100 cells/µL, given that 16% of all CD4 tests done in our population are less than or equal to 100 cells/µL and the vast majority of low CD4 counts are for newly diagnosed patients. For the base model, with a CRAG prevalence of 0.0906 amongst those with a CD4<100 cells/µL, and presumed 16% with a CD4<100 cells/µL, CrAg prevalence amongst all those in an HIV test-and-treat program was 0.0145. This assumes that CrAg prevalence is 0% among those with higher CD4 cells counts.

In Figure 2a, for those with a new HIV diagnosis, if a CrAg test is conducted, the result is either CrAg negative or CrAg positive. CrAg positive persons are again disaggregated into three categories: (1) asymptomatic and thus eligible for preemptive treatment with fluconazole; (2) symptomatic with meningitis who should be hospitalized; and (3) CrAg positive due to a history of cryptococcal infection. CrAg negative persons and those with a history of cryptococcal infection start ART immediately.

In this model, there is a subgroup that was not CrAg tested and immediately started on ART (**Figure 2a**). This is a realistic assumption given advocacy for immediate ART in all persons diagnosed with HIV infection by the WHO and the absence of guidelines for CrAg screening without CD4 testing. Thus, there are CrAg positive persons (both asymptomatic and symptomatic) who are not preemptively treated for cryptococcal infection. This group has a high risk of meningitis and/or unmasking immune reconstitution inflammatory syndrome (IRIS) when initiating ART. The risk of developing meningitis and IRIS amongst CrAg positive persons has not been evaluated previously. We know from prior studies that those who are asymptomatic CrAg positive treated with ART but not fluconazole have very high mortality however.(6) We assumed progression to meningitis or IRIS having received ART but not fluconazole for *symptomatic* CrAg positive persons was 100%. Those *asymptomatic* CrAg positive have a 50% probability of progressing to CM or IRIS with ART but no fluconazole. While it is possible in the CD4 based model that persons are started on ART and not CrAg screened, we assume that the majority of death in the CD4 based model occurs early, due to lack of ART and lack of preemptive treatment for cryptococcal infection. In the test-and-treat model, we assume ART is initiated more rapidly; there are less delays in initiating ART, but there is still poor outcome from untreated cryptococcal infection.

Asymptomatic CrAg positive persons are categorized as having a high titer (>1:160), therefore more likely to progress to meningitis and/or death, or having a lower titer (<1:160) therefore less likely to develop meningitis or death (**Figure 2b**). For those asymptomatic CrAg positive, and thus eligible for preemptive treatment, it is assumed that all are treated with preemptive fluconazole. Risk of progression to meningitis is the same as described above in Figure 1b. Outcomes for asymptomatic CrAg positive persons who receive fluconazole are the same as those described in the above strategy of CrAg screening for those with CD4<100 cells/µL. It is presumed that those who were CrAg positive, initiate ART 2 to 4 weeks after starting antifungal therapy per WHO recommendations.(7)

For those with meningitis (whether at the time of screening, or among those who failed preemptive treatment, or those symptomatic CrAg positive persons), probability of hospitalization is the same as described above in figure 1c. Of those hospitalized, mortality from cryptococcal IRIS is estimated to be 50%.(8)

References

1. Morawski B, DR; B, al. e. Pre-ART Cryptococcal Antigen Titer Associated with Preemptive Fluconazole Failure. CROI. 2016.

2. Molloy S KC, Heyderman R, Loyse A, Kouanfack C, Chanda D, Mfinanga S, Temfack E, Lakhi S, Lesikari S, Chan A, Stone N, Kalata N, Karunaharan N, Gaskell K, Peirse M, Ellis J, Chawinga C, Lontsi S, Ndong JG, Bright P, Lupiya D, Chen T, Bradley J, Adams J, van der Horst C, Oosterhout JJ, Sini V, Mapoure YN, Mwaba P, Bicanic T, Lalloo D, Wang D, Hosseinipour M, Lortholary O, Jaffar S, Harrison T, ACTA Trial Study Team. , editor A randomized controlled trial for the treatment of HIV-associated cryptococcal meningitis in Africa: oral fluconazole plus flucytosine or one week amphotericin-based therapy vs two weeks amphotericin-based therapy. The ACTA Trial. International AIDS Society; 2017; Paris, France.

3. Butler EK, Boulware DR, Bohjanen PR, Meya DB. Long term 5-year survival of persons with cryptococcal meningitis or asymptomatic subclinical antigenemia in Uganda. PloS one. 2012;7(12):e51291.

4. International Medical Products Price Guide Arlington, VA: Management Sciences of Health; 2016 [Available from: <http://mshpriceguide.org/en/home/>.

5. Organization WH. Guidelines for the diagnosis, prevention, and management of cryptococcal disease in HIV-infected adults, adolescents and children. . 2018.

6. Meya DB, Manabe YC, Castelnuovo B, Cook BA, Elbireer AM, Kambugu A, et al. Cost-effectiveness of serum cryptococcal antigen screening to prevent deaths among HIV-infected persons with a CD4+ cell count < or = 100 cells/microL who start HIV therapy in resource-limited settings. Clinical infectious diseases : an official publication of the Infectious Diseases Society of America. 2010;51(4):448-55.

7. World Health Organization. Guidelines for Managing Advanced HIV Disease and Rapid Initiation of Antiretroviral Therapy 2017 [Available from: <http://apps.who.int/iris/bitstream/10665/255884/1/9789241550062-eng.pdf?ua=1>.

8. Rhein J, Hullsiek KH, Bahr NC, Tugume L, Nuwagira E, Ssebambulidde K, Kiggundu R, Evans EE, Mpoza E, Williams DA, Abassia M, Musubire A, Muzoora C, Meya DB, Boulware DR, editor High Mortality Associated with Unmasking Cryptococcal Meningitis. CROI; 2018; Boston.
